# Special Issue “Corrosion Resistance of Alloy and Coating Materials”

**DOI:** 10.3390/ma15176127

**Published:** 2022-09-03

**Authors:** Zbigniew Brytan

**Affiliations:** Department of Engineering Materials and Biomaterials, Faculty of Mechanical Engineering, Silesian University of Technology, Konarskiego 18a, 44-100 Gliwice, Poland; zbigniew.brytan@polsl.pl

This Special Issue aims to include the latest research findings on the corrosion phenomena that occur in various materials, both solid and coating materials. The works cover several exciting aspects related to corrosion of magnesium, titanium, aluminium alloys, and High-strength low-alloy steel (HSLA). It also refers to coating materials applied by various techniques, including high velocity oxygen fuel (HVOF), air plasma spraying (APS), atomic layer deposition (ALD), physical vapor deposition (PVD), and their subsequent processing by laser remelting. The scope of the presented works is extensive and multidisciplinary, but still oriented towards one axis, which is corrosion and its research.

The study of protective film formation on Mg alloys by exposing them to sodium selenite solutions revealed that the protective film formed on the AZ31 alloy provided a five-fold increase in corrosion resistance [1]. In the study [2], the researchers fabricated casein-coated, casein-free-coated, and uncoated Mg alloys. MgCaZn1 and MgCa2Zn1Gd3. Coated samples of both alloys exhibited a higher corrosion potential, a high polarization potential, and a lower volume of H_2_ release than the corresponding uncoated alloy. On the basis of the same parameters, it is evident that the casein-coated Gd-doped Mg alloy exhibited better corrosion resistance than the standard Mg-Ca-Zn alloy.

The nickel-based alloys are also in the scope of collected papers. Nickel-based superalloy was investigated under pre-oxidation conditions to verify the influence of the scale formed during the hot corrosion phenomena [3]. The study [4] focuses on the corrosion behavior of nickel superalloy coated with Cr_3_C_2_-25NiCr powder through APS and HVOF at 950 °C in environments of air oxidation and molten salt. The HVOF process has been shown to provide better coating characteristics in terms of bond strength and high-temperature corrosion resistance in both environments compared to the APS process. Meanwhile, the study in [5] focuses on evaluating the corrosion resistance of NiTi (Nitinol) wire through the heat treatment and the passivation process. In summary, it is preferable that the heat treatment process is carried out in a salt furnace rather than an air furnace, and it is suggested that the corrosion resistance can be improved by going through a subsequent passivation process.

Research on titanium alloys for biomedical applications revealed that the Ti6Al4V alloy with the ZnO layer deposited by the ALD process shows an improvement in the physicochemical properties of Ringer solution [6]. In the paper [7], researchers investigate the electrochemical deposition process of Cu-Sn-TiO_2_ coatings from a sulfuric acid electrolyte. The addition of TiO_2_ significantly improved the antibacterial properties of the composites. In the paper [8], interesting studies on the modification of Zn-based protective coatings by tungsten trioxide (WO_3_) nanoparticles were presented. The effect of heat treatment on the corrosion resistance of newly developed HSLA-type steel 0.28C-1.40Mn-0.3Si-0.26Cr with micro-additions of Nb, Ti, and V was discussed in the article [9].

The eutectic high-entropy alloys (EHEAs) like AlCoCrFeNi_2.1_ is also in the scope of the Special Issue. In the work [10] corrosion behavior of the AlCoCrFeNi_2.1_ in chloride-containing sulfuric acid solution at different temperatures were studied. The effects of temperature on the passive and local corrosion mechanism of EHEA were discussed. Corrosion of the EHEA occurs preferentially in the Al-Ni-rich phase in the chloride-containing sulfuric acid solution. Pitting is the main form of corrosion at low temperatures, while both pitting and selective dissolution occur at a higher temperature. 

The issue also contains works related to laser surface treatment of materials. The cermet coating Cr_3_C_2_-25(Ni20Cr) on the aluminium alloy Al7075 (EN, AW-7075) deposited by cold spraying was subsequently laser remelted [11]. The laser remelting caused homogenisation of the cermet coatings structure and eliminated microcracks and pores on their surface. It was found that the corrosion rate of the coating can be reduced more than two-fold when the optimal processing condition is applied. The research presented in [12] evaluates properties of NiCr(Ti) protective coatings on mild steel plates prepared by diode laser cladding technology. The NiCr coatings with different Ti additions are intended to be used as potential materials for bipolar plates in polymer electrolyte membrane fuel cells. Presented results shows that coatings with 5 - 10%Ti addition comply with the conditions of the US Department of Energy (US DOE) regarding corrosion performance to be used as materials for the manufacture of the bipolar plates.

The studies of laser beam-welded (LBW) joints in various configurations of a CoCrMoW alloy used in prosthodontics with nickel-free stainless steel and cobalt alloy wire are presented in [13]. The influence of filler metal composition on corrosion characteristics was tested in artificial saliva environment. Authors concluded that it may be acceptable to use stainless steel wire instead of CoCr for welding CoCrMoW dental alloys and such an interchangeable use of that filler material does not create a strong corrosive cell in artificial saliva.

Hybrid techniques for the production of coatings and the assessment of their corrosion resistance are also presented. The article [14] shows the synergy of the effect of two surface engineering technologies: magneto-sputtering (MS-PVD) and atomic layer deposition (ALD) on the structure and properties of the bimodal TiO_2_/nanoTiO_2_ coating deposited on the stainless steel substrate 316L.

The innovative research methodology was also included in the scope of the presented works. The relationship between the degradation of the coating of gas turbine blades and its surface color was discussed in the paper [15]. The proposed approach significantly expands the possibilities of the nondestructive appraisal of gas turbine blades, especially in assessing their technical state without the need for their disassembly. In practical applications, this solution will provide a credible method for gauging the degradation state of gas turbine blades based on changes in their surface color.

The Editors give special thanks to the authors and the editorial team of *Materials* for the collaborative and peer-review process. We hope you enjoy reading this Special Issue and find new concepts for future and present research works.

## Data Availability

Not applicable.
